# Age-Related Changes and Sex-Related Differences in Brain Iron Metabolism

**DOI:** 10.3390/nu12092601

**Published:** 2020-08-27

**Authors:** Tanja Grubić Kezele, Božena Ćurko-Cofek

**Affiliations:** 1 Department of Physiology and Immunology, Faculty of Medicine, University of Rijeka, Braće Branchetta 20, 51000 Rijeka, Croatia; bozena.curko.cofek@medri.uniri.hr; 2 Clinical Department for Clinical Microbiology, Clinical Hospital Center Rijeka, Krešimirova 42, 51000 Rijeka, Croatia

**Keywords:** aging, Alzheimer’s disease, iron metabolism, multiple sclerosis, Parkinson’s disease, sex differences, stroke

## Abstract

Iron is an essential element that participates in numerous cellular processes. Any disruption of iron homeostasis leads to either iron deficiency or iron overload, which can be detrimental for humans’ health, especially in elderly. Each of these changes contributes to the faster development of many neurological disorders or stimulates progression of already present diseases. Age-related cellular and molecular alterations in iron metabolism can also lead to iron dyshomeostasis and deposition. Iron deposits can contribute to the development of inflammation, abnormal protein aggregation, and degeneration in the central nervous system (CNS), leading to the progressive decline in cognitive processes, contributing to pathophysiology of stroke and dysfunctions of body metabolism. Besides, since iron plays an important role in both neuroprotection and neurodegeneration, dietary iron homeostasis should be considered with caution. Recently, there has been increased interest in sex-related differences in iron metabolism and iron homeostasis. These differences have not yet been fully elucidated. In this review we will discuss the latest discoveries in iron metabolism, age-related changes, along with the sex differences in iron content in serum and brain, within the healthy aging population and in neurological disorders such as multiple sclerosis, Parkinson’s disease, Alzheimer’s disease, and stroke.

## 1. Introduction

Iron is an essential micronutrient because of its importance in the process of erythropoiesis, oxidative metabolism, and cellular immune responses [1]. Healthy adults contain 4–5 g of iron, which is mostly (65%) found in red blood cell hemoglobin (Hb), and 30–35% is stored in the liver in the form of ferritin. Only 1–2% is in the form of iron-sulfur clusters or heme in the enzymes and multiprotein complexes [2]. Despite its essential role in the human body [3], there are no effective means of excreting iron [4]. Thus, a critical point in iron homeostasis is the regulation of the absorption of dietary iron from the duodenum. The body absorbs 1–2 mg of dietary iron a day, and this intake must be balanced with losses in the form of sloughed intestinal mucosal cells, menstruation, and other blood losses [5]. Maintaining the balance is very important because free iron is able to generate free radicals through Fenton reaction, and it is highly toxic [6,7]. Therefore, organisms have developed sophisticated pathways to import, chaperone, sequester, and export iron in order to maintain an appropriate iron balance [8]. Any disruption of iron homeostasis leads to either iron deficiency (ID) or iron overload (IO) [9]. 

The pathophysiological consequences of ID are impairments in cognitive development, cardiovascular diseases, endothelial disorders, and other health complications [10], especially in elderly. A chronic low-grade inflammation present in the elderly leads to less efficient absorption through hepcidin regulation and subsequent increase in ferritin concentrations. ID becomes more of a problem because of age-related changes in Hb and sex hormones, effects of medication prescribed for age-related diseases, and metabolic changes associated with inflammatory states [11]. 

IO leads to adverse manifestations in different tissues (brain, heart, liver, adipose, muscle, pancreas) and it is implicated in the pathogenesis of several metabolic (e.g., type 2 diabetes, non-alcoholic steatohepatitis, atherosclerosis, stroke, etc.) [12] and neurodegenerative diseases (e.g., Alzheimer’s disease (AD), Parkinson’s disease (PD), multiple sclerosis (MS), etc.) [13], which could be found more often in elderly.

Although physiological iron requirements do not differ between adult and elderly men, and post-menopausal and elderly women, there is growing evidence that iron metabolism is affected by the aging process [11]. Age-related cellular and molecular alterations in iron metabolism can lead to iron dyshomeostasis and deposition [14]. Iron deposits can contribute to the development of inflammation, abnormal protein aggregation and degeneration, especially in the central nervous system (CNS), leading to the progressive decline in cognitive processes [13], and contributing to pathophysiology of stroke [15]. 

An increased number of recent experimental and clinical discoveries about the sex-related differences in iron metabolism present in certain neurological disorders [16,17,18,19,20,21,22] is raising attention, and pointing to the need for future research exploring possible underlying mechanisms, which could be responsible for the sex differences in the susceptibility to these diseases.

Whereas environmental factors are involved in most of the cases of neurodegenerative diseases, it is important to take into consideration the role of nutrition in both neuroprotection and neurodegeneration. Furthermore, there is sufficient evidence regarding ID having adverse health effects to justify correcting it through diet or iron therapy. At the same time, it is important to ensure that the risk of high body iron stores is not increased as this may have detrimental effects on the brain and cause neurodegeneration and other neurological disorders [23,24]. Therefore, the aim of this review is to provide an up-to-date discussion in iron metabolism, age-related changes, along with the sex differences in iron content in serum and brain, within the healthy aging population and in neurological disorders such as MS, PD, AD, and stroke.

## 2. Systemic Iron Metabolism

In the last 15 years, many new iron genes, proteins and pathways have been discovered thanks to the application of genetic screens and transgenic technology in biomedical research. They brought a new light in the understanding of iron absorption, trafficking, utilization, and regulation [25].

### 2.1. Iron Absorption

All iron enters the body from the diet. Normal diet should contain 13–18 mg of iron per day but only 1–2 mg is absorbed [26]. Absorption of dietary iron mainly occurs at the apical surface of the mature duodenal enterocytes [27]. Low pH of duodenum favors solubility of iron while further down the intestine the formation of insoluble ferric complexes probably reduces bioavailability [26]. It is well known that vitamin C enhances iron uptake in the duodenum but it is also important at cellular level since it enables efficient uptake of iron from transferrin, which is the only source of iron for erythropoiesis [28].

Dietary iron can be in heme (10%) and non-heme (ionic, 90%) forms and they absorb via different mechanisms [26]. Dietary non-heme iron primarily exists in an oxidized (Fe^3+^) ferric form that is not bioavailable, and therefore must be reduced to the ferrous (Fe^2+^) form before the transport across the intestinal epithelium [29,30]. The reduction of iron from the ferric to the ferrous state occurs at the enterocyte brush border by a duodenal cytochrome b (DCYTB), an iron-regulated ferric reductase enzyme [27,29].

Ferrous iron is then transported across the apical plasma membrane of the enterocyte by divalent metal transporter-1 (DMT1) [31,32]. Inside the enterocyte, iron may be stored as ferritin and excreted in the faeces when the senescent enterocyte is sloughed. The other possibility is the transfer of iron out of the enterocyte across the basolateral membrane to the plasma by the basolateral iron exporter ferroportin-1 (FPN1) [33,34]. FPN1 is a 12 transmembrane domain protein (also known as SLC40A1, IREG1, or MTP1) [35]. It transports ferrous iron, which must be oxidized by a multi-copper oxidase protein called hephaestin (HEPH) to ferric iron [30,36], which binds to plasma transferrin (Tf) [37,38].

The research supports the existence of transferrin-independent routes of iron uptake. DMT1 was thought to be responsible for non-transferrin-bound iron (NTBI) uptake by liver cells, but iron loading of DMT1-deficient mouse hepatocytes indicates that there is at least one alternative transferrin-independent uptake pathway [39]. The main candidate for this alternative pathway is a zinc transporter Zrt–Irt-like protein 14 (ZIP14, SLC39A14). It is considered as the second major carrier involved in NTBI uptake by hepatocytes [40].

Heme iron is absorbed into enterocytes by a heme carrier protein 1 (HCP1). It is a membrane protein in the proximal intestine, where heme absorption is the greatest [41,42]. Once internalized in the enterocytes, the most dietary heme iron is released as ferrous iron by heme oxygenase (HO) and enters a common pathway with dietary non-heme iron before it leaves the enterocytes [1]. It remains unclear whether some heme traverses the cells intact and leaves the enterocytes through the action of the heme exporters breast cancer resistance protein/ATP-binding cassette subfamily G member 2 (BCRP/ABCG2) and feline leukemia virus C receptor (FLVCR) [41]. 

### 2.2. Iron Transport and Distribution

Except for the duodenal enterocytes, the sources of iron in the plasma are iron-recycling reticuloendothelial macrophages [43]. When iron enters the circulation, it binds to Tf and is transported to sites of use and storage [44]. Human Tf is a 76-kDa glycoprotein mainly produced in the liver. One Tf molecule can carry two ferric ions. Serum Tf can be in the non-iron bind (apo-Tf), monoferric, or diferric (holo-Tf) forms [45,46].

The interaction between Fe^3+^ and Tf is dependent on pH. Fe^3+^ efficiently binds to Tf at pH 7.4 and dissociates from Tf at acidic pH [46]. Iron enters cells that require iron through a transferrin receptor 1 (TfR1)-mediated mechanism [47]. Holo-Tf has a much higher affinity for TfR than monoferric-Tf. Therefore, holo-Tf binds to TfR1 on the cell surface and the whole complex is internalized by clathrin-mediated endocytosis forming clathrin-coated endosomes (siderosomes) [44]. In the acidic endosomal milieu, Fe^3+^ dissociates from Tf and is converted to Fe^2+^ by metalloreductases [48]. Fe^2+^ is transported into the cytosol by DMT1 while Tf/TfR1 complex is transported to the cell surface and both elements are reused in another cycle of cellular iron uptake [46]. TfR1 is expressed in all tissues [49] and its expression is regulated by the cellular iron status at both the transcriptional and post-transcriptional levels. In the presence of hypoxia or ID, the expression of hypoxia-inducible factors (HIF-1α and HIF-2α) increases, and these proteins bind to the hypoxia response element (HRE) in the promoter of transferrin receptor (TFRC) gene, thereby promoting TFRC transcription [50]. At the post-transcriptional level, the iron regulatory protein (IRP)/iron responsive element (IRE) system plays an important role [51]. Under intracellular iron-deficient conditions, intracellular iron sensors IRP1 and IRP2 bind to IREs to stabilize the TFRC mRNA and enhance the expression of TfR1 protein. In the case of iron excess, IRPs lose their interactions with IREs. IRP1 becomes an aconitase through conformational changes [52] and IRP2 is degraded after ubiquitination [53,54], resulting in destabilization and degradation of TFRC mRNA. 

Transferrin receptor 2 (TfR2) mRNA is highly expressed in the liver and to a lesser extent in spleen, lung, muscle, prostate and peripheral blood mononuclear cells [46,55]. It binds to holo-Tf with 20-fold lower affinity than TfR1 [56]. Since TfR2 lacks an IRE, it is not regulated in response to the plasma iron level. Instead, TfR2 expression is reciprocally regulated by Tf saturation, and consequently upregulated in IO [5]. It was also suggested that TfR2 could be involved in the uptake of NTBI [57].

### 2.3. Iron Storage and Recycling

The liver is the main storage site for excess iron [49]. The uptake of Tf-bound iron from plasma to liver cells is through the TfR1 and TfR2. Since free intracellular iron is toxic, most of the iron in the cells is stored in ferritin [58]. The poly(rC)-binding proteins act as intracellular iron chaperones and deliver iron to ferritin and several enzymes [59].

Ferritin is the main form of iron storage and can be found in all tissues, but mostly in the liver, spleen, and in bone marrow. It has the capacity to sequester up to 4500 atoms of non-heme iron in its spherical structure [60]. This spherical structure consists of 24 subunits of heavy (H) and light (L) ferritin in different ratios, depending on the tissue [61]. Within the ferritin, iron is stored in the ferric form [62]. Ferritin shows enzymatic properties by converting ferric to ferrous iron as iron is sequestered in the ferritin mineral core [63]. When high concentrations of iron-laden ferritin accumulate within the cell, the ferritin molecules aggregate, and fuse with lysosomes. This process leads to the degradation of ferritin, and the resulting mixture of Fe^3+^ cores and peptides is known as hemosiderin [64]. Iron can be efficiently mobilized from both ferritin and hemosiderin when it is required elsewhere in the body.

Another important feature of ferritin is that small amounts are secreted from the cell, and this amount strongly correlates with the concentration of intracellular iron. This association makes serum ferritin concentrations an accurate indicator of body iron stores [65].

Mitochondrial ferritin is also an iron-storage protein. Its amino acid sequence shares high homology with H-chain ferritin, indicating similar functions [66,67]. It has been shown that mitochondrial ferritin expression is limited to tissues with high metabolic activity and oxygen consumption, such as brain, testis, and heart [68]. 

A significant part of iron (600 mg) is deposited in tissue macrophages [69], which respond to systemic iron requirements by the interaction of hepcidin and FPN1 [70,71]. Iron storage at the macrophages is safe and does not lead to oxidative damage [72]. The amount of iron required for daily production of red blood cells (20–30 mg) is provided mostly by iron recycling by macrophages [1,73]. Splenic and hepatic macrophages phagocytize and degrade senescent and damaged erythrocytes to recover iron, mainly to produce Hb in new erythrocytes but also for other carriers and enzymes requiring iron [69]. In the phagocytic vesicles, heme is metabolized by HO and iron is exported to the cytoplasm by a protein similar to DMT1 [1].

Erythropoietin reduces iron retention in macrophages by decreasing DMT1 and increasing FPN1 expression [72]. Macrophages obtain a certain amount of iron from plasma through the action of DMT1 and TfR1, and from apoptotic cells and bacteria [44]. 

### 2.4. Regulation of Systemic Iron Homeostasis

All the processes involved in maintaining iron homeostasis are regulated at the different levels, mainly by the interaction and cooperation of three systems [74]. The first system consists of hormone hepcidin and iron export protein FPN1. They act on systemic level and regulate serum iron levels [75]. The post-transcriptional regulation of iron genes involved in intracellular iron homeostasis is mediated by the interaction of IRPs and IRE while HIF2 α mediates transcriptional regulation of iron homeostasis [76]. 

Hepcidin is a key regulator of iron level. Variations in body iron demand are communicated to the liver which, in turn, modulates the expression of hepcidin [77], encoded by the hepcidin antimicrobial peptide gene (HAMP) [78]. Hepcidin is primarily produced by liver sinusoidal endothelial cells in response to iron levels [75,79], and in the small quantity by macrophages [80] and adipocytes [81]. Different physiological and pathological conditions such as increased erythropoiesis, hypoxia, anemia, IO, endocrine, metabolic, and inflammatory processes affect hepcidin synthesis in hepatocytes [63,82]. Hepcidin is upregulated in response to iron loading and inflammation and decreases in response to ID and hypoxia [83,84]. 

Hepcidin transcription is regulated by bone morphogenic protein 6 (BMP6) [85], which acts on hepatocytes through BMP receptor (BMPR) [86]. BMPR creates a supercomplex with hemojuvelin (HJV), matriptase 2 (MT2) and neogenin [87].

Activated BMPR induces phosphorylation of s-mothers against decapentaplegic (SMAD) molecules, which then cause an increase in hepcidin expression through activation of HAMP gene [38]. Tumor necrosis factor (TNF), pathogens, and interleukin-6 (IL-6) stimulate hepcidin synthesis via signal transducer and activator of transcription 3 (STAT-3) activation [77,88]. Hepcidin expression in macrophages is regulated mainly through toll-like receptor 4 (TLR4) associated with adaptor proteins [89].

Iron sensing is dependent on an interaction of Tf, TfR1 and TfR2, aided by the hemochromatosis protein (HFE). HFE has an extracellular transferrin receptor-binding region and forms a stable complex with TfR1 [90]. When HFE binds to TfR1, HFE changes the conformation of the Tf-Fe binding site, decreasing the affinity of TfR1 for Tf and iron entry into cells [49,84,91]. 

Hepcidin expression decreases the iron absorption from the duodenal enterocytes, iron release from macrophages and its transport across the placenta [92,93]. The iron exporter required for iron egress from enterocytes, macrophages, as well as all other iron exporting cells including placental syncytiotrophoblasts and hepatocytes, is FPN1. It is not only the effector of cellular iron export, but also the receptor for hepcidin, its primary regulator [94]. Hepcidin binds to FPN1 present on the cell surface and induces the phosphorylation of amino acids located on an intracellular loop of FPN1, triggering the internalization of the hepcidin-FPN1 complex, leading to the ubiquitination of FPN1 and lysosomal degradation of both proteins [65]. 

In the inflammatory conditions, upregulation of hepcidin is mainly through IL-6 [93,95], which induces STAT-3 activation and its binding to the hepcidin promoter [96]. The increased hepcidin synthesis causes iron sequestration in macrophages and decreases iron availability in tissues, limiting the growth of microbes [70] and causing the characteristic hypoferremia and eventually anemia of inflammation [93,95].

## 3. Brain Iron Metabolism

The brain is a very metabolically active organ and accounts for about 20% of the body’s total energy consumption. These high-energy needs must be supported with an adequate supply of iron [97]. Therefore, iron is the most abundant metal in the brain [14]. It has an essential role as a co-factor for many physiological processes in the CNS, including oxidative metabolism, myelination, and the biosynthesis of neurotransmitters [98]. To ensure the normal course of these processes, brain iron levels are tightly regulated [99]. 

**Figure 1:**
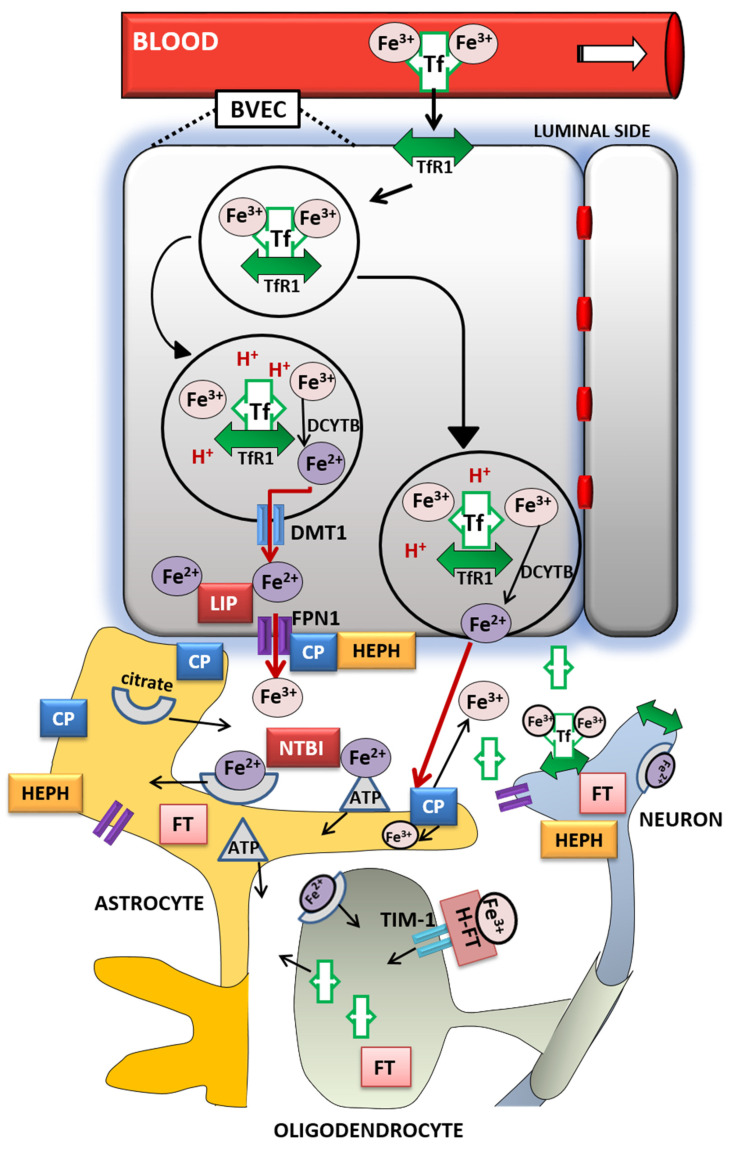
Iron transport inside the brain. A scheme of proposed transferrin-bound and non-transferrin bound iron transport pathways in the brain. *Abbreviations*: BVEC—blood vascular endothelial cell, Tf—transferrin, TfR1—transferrin receptor 1, Fe^3+^—ferric iron, Fe^2+^—ferrous iron, DCYTB—duodenal cytochrome b, DMT1—divalent metal transporter-1, FPN1—ferroportin-1, LIP—labile iron pool, CP—ceruloplasmin, HEPH—hephaestin, NTBI—non-transferrin-bound iron, FT—ferritin, H-FT—H-ferritin, TIM-1—T-cell immunoglobulin and mucin domain.

The entry of iron from the blood into the brain is controlled by the blood–brain barrier (BBB) [100] and to a lesser extent by the blood–cerebrospinal fluid barrier (BCSFB) [101]. The role of the BBB is to prevent the brain from neurotoxic plasma components and pathogens [102]. At the same time, it controls chemical composition of the neuronal milieu by regulating the transport of molecules required for normal neuronal functioning [103]. The BBB is formed by a monolayer of tightly sealed microvascular endothelial cells extending along the vascular tree [104] and expressing low paracellular and transcellular permeability [105]. Those endothelial cells are surrounded by basal lamina and astrocytic perivascular end-feet, forming the neurovascular unit [106]. Tf-bound iron cannot cross the BBB directly and the mechanism of iron transcellular entry into the brain is not entirely clear [107]. According to the recent models [108,109], there are two possible iron transport pathways: transferrin-bound iron (Tf-Fe) and NTBI [107] (Figure 1). 

The Tf/TfR1 pathway is considered to be the major route for iron transport across the luminal membrane of the capillary endothelium [107]. According to the widely established transcytosis mechanism, Tf binds to TfR at the luminal side of the brain capillaries [32]. The complex traverses the cell in the endocytosis vesicle, where the acid environment facilitates the release of ferric iron from Tf and its reduction to ferrous iron by endosomal reductase [110], possibly DCYTB or six-transmembrane epithelial antigen of the prostate-2 (STEAP2) [111]. The next steps in this pathway are still not completely clear. One possibility is that ferrous iron is transported from the endosome to the cytosol by the DMT1 [112] and joins the intracellular labile iron pool (LIP) [113] (Figure 1). It could be further utilized for metabolic purposes by the endothelial cells, stored in endothelial cell ferritin [114] or imported into mitochondria via mitoferrins and TfR2 [115]. It could be also released into the extracellular fluid by action of export protein FPN1 [108], and reoxidized to Fe^3+^ by ferroxidases HEPH and ceruloplasmin (CP) [100]. Studies have confirmed that capillary endothelium of the BBB, neurons, and astrocytes, has the ability to express FPN1 and HEPH [116,117]. The alternative mechanism that has been proposed is that the endosome containing Tf-TfR1 complex goes all the way to the abluminal side and releases iron between the endothelial cells and astrocyte end-foot processes [99]. The released ferrous iron is then oxidized to ferric iron by the ferroxidase activity of CP or HEPH expressed on the end-foot processes [112]. Oxidized iron binds to apo-Tf circulating within the brain [113] (Figure 1). The main source of Tf in the brain interstitium is its diffusion from the ventricles and a certain amount is synthesized in oligodendrocytes [118]. Because of the low concentrations of Tf in the cerebrospinal fluid (CSF), iron saturation of CSF Tf is almost 100%, while serum Tf is saturated by about 30% [99]. Consequently, under conditions of IO, CSF Tf has much lower buffering capacity [119], NTBI levels may be quite high [120], and the vulnerability of neuronal cells to iron toxicity increases [119]. 

Iron may also enter the brain through epithelial cells of the choroid plexus, which form the BCSFB [121]. The choroid plexus consists of fenestrated capillaries so the holo-Tf can cross them and reach the choroidal epithelium [122]. Further, the iron is released the same way as from the BBB endothelial cells by means of DMT1, FPN1 and ferroxidases [14]. When iron enters the CSF, there is no diffusional barrier between CSF and interstitial fluid. Iron binds to Tf in CSF and supplies CNS cells expressing TfR1 [123]. 

Different cell types in the brain acquire iron by distinct pathways. Neurons express high levels of TfR1. Therefore, Tf is the main source of iron for neurons [112], although neurons can also uptake NTBI from interstitial fluid [124]. Unlike them, oligodendrocytes and astrocytes do not express TfR1 and their main source of iron is NTBI [110]. Namely, ferrous iron in the brain interstitium can also bind to ATP or citrate released from astrocytes and it is transported to oligodendrocytes and astrocytes as NTBI [99] (Figure 1).

Oligodendrocytes acquire NTBI via the T-cell immunoglobulin and mucin domain (Tim-1). It is a ferritin receptor exclusively expressed in oligodendrocytes that binds H-ferritin [125]. Astrocytes express ferri-reductase on their plasma membranes to reduce ferric to ferrous iron and facilitate iron uptake [126] (Figure 1). Once iron enters the brain cells, the iron pool is tightly regulated. It has to provide enough iron for cellular functions and prevent the development of oxidative stress [110]. Ferritin has an important role in iron sequestration and free iron level reduction [127], whereas neuromelanin captures large amounts of iron in certain neurons for longer-term storage [128]. Namely, the pigment neuromelanin acts as a scavenger binding redox-active metal ion such as iron. The expression of ferritin varies in different cell types according to their functional requirements for iron. Neurons contain the least, and microglia contain the most amount of cytosolic ferritin [129] but in the hypoxic conditions ferritin synthesis increases in cortical neurons and decreases in glial cells [130]. Ferritin degradation by the autophagy-lysosome system [131] initiates iron release, mainly through FPN1 [132]. Since hepcidin regulates the expression of FPN1, it modulates cellular iron level as well [13]. Recent studies revealed that hepcidin can be produced by the brain endothelium [108] or systemically derived by passing the BBB [133], and it is widely distributed in the brain [134,135]. Hence, hepcidin may be involved in the regulation of iron availability and circulation in the brain [108]. Cellular iron levels are also modulated at the post-transcriptional level by binding to the IREs of mRNA of IRPs [13].

When some of these cellular and molecular mechanisms of iron regulation are disrupted, the brain iron homeostasis is disturbed as well. If there is either too much or too little iron in the brain, numerous neurologic disorders can occur [14]. Excessive brain iron accumulation is found in MS, PD and AD, amyotrophic lateral sclerosis (ALS), neurodegeneration with brain iron accumulation, and Huntington’s disease [114]. ID is associated with significant cognitive, performance and brain structural deficits [136]. 

## 4. Age-Related Iron Dyshomeostasis

**Figure 2:**
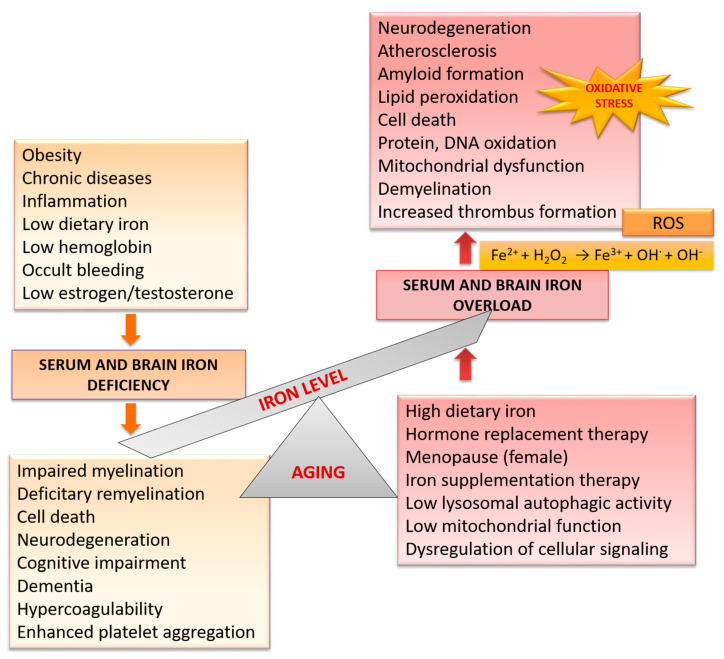
Age-related changes in iron level and consequent brain disorders.

Many iron homeostatic mechanisms appear to be affected during physiological aging (Figure 2). Therefore, older age is associated with increased risk of ID, elevated body iron stores and increased brain iron levels [23]. 

Iron deficiency is the most common nutrient deficiency worldwide [137]. The causes underlying ID, i.e., ID anemia are diverse and include: inadequate oral iron intake resulting from poor diets, excessive milk intake or vegetarian diets, inadequate iron absorption as a result of celiac disease and others, or excessive iron loss, mainly because of the blood loss or as a result of parasitic infection [137]. Inadequate iron supply leads to cerebral hypoxia [138], insufficient neurotransmitter synthesis [13], poor myelin integrity [139,140], and consequently to poor cognition, cognitive decline, and dementia [141].

Body iron levels may be elevated in older adults due to consumption of highly bioavailable forms of iron, such as supplemental iron and red meat, or enhancers of nonheme-iron absorption like vitamin C [142]. Studies have shown that high body iron stores were associated with increased risk of coronary heart disease [143], type 2 diabetes [144], and cognitive impairment and dementia [145]. 

Brain iron accumulation is considered as a hallmark of aging [146] and it is associated with the progressive imbalance between antioxidant defenses and intracellular generation of reactive oxygen species (ROS) [147]. This may explain the increased susceptibility of the aged brain to disease and the reason why aging is the major risk factor in neurodegenerative diseases [14].

Post-mortem analyses showed a positive correlation between iron deposition and age as well as the different iron contents in different brain regions [148]. Iron staining in older individuals (60–90 years of age) showed a larger content of iron in the microglia and astrocytes of the cortex, hippocampus, cerebellum, basal ganglia, and amygdala [149,150].

Increase in iron concentration, in the form of H- and L-ferritin, occurs in the *substantia nigra* [114] with many extraneuronal iron deposits in individuals over 80 years of age, especially in oligodendrocytes. Neuronal deposits in the *substantia nigra* are found in the neurons that do not contain neuromelanin [151]. Modern non-invasive methods, such as magnetic resonance imaging (MRI), revealed an age-related increase in the non-heme iron concentration in the *nucleus caudatus, putamen* and *globus pallidus* [152,153]. 

The mechanism of increase in iron concentration in certain brain regions is not completely clear. One of the proposed mechanisms is altered vascularization. It is observed during aging and in neurodegenerative diseases [154]. Region-specific increase of total iron could be probably triggered by inflammation [155], increased BBB permeability [156], redistribution of iron within the brain, and changes in the iron homeostasis [13]. The increase in iron concentration inside the CNS cells might directly damage these cells or affect the cellular environment, making it more susceptible to toxins and activation of pathogenic processes [151]. Besides, during brain aging, iron is partially converted from its stable and soluble form (ferritin) into hemosiderin and other oxyhydroxides that contain iron at higher reactivity [157], inducing the neuronal vulnerability to oxidative stress [158]. An additional feature of brain aging that contributes to the development of oxidative stress is an increase in the levels of monoamine oxidase (MAO). This enzyme catalyzes the oxidative deamination of neurotransmitters, in which hydrogen peroxide (H_2_O_2_) and aldehydes as highly toxic by-products are subsequently generated [159]. Since those by-products are inductors of lipid peroxidation, it is assumed that activation of MAO is associated with age-related disturbances of the homeostasis and generation of free radicals in the nervous tissue [160]. 

## 5. Sex-Related Differences in Iron Homeostasis during Healthy Aging and in Neurological Disorders

**Figure 3:**
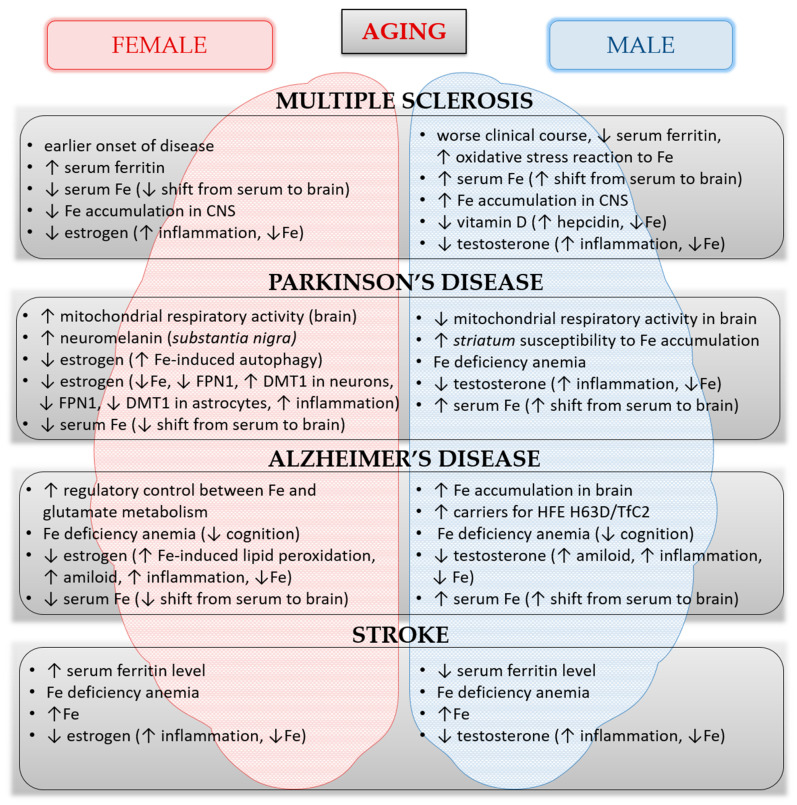
The summary of features that involve iron impact on increased susceptibility to certain neurological disorders or increased progression of already present disorders in males and females during aging. The summary includes data from the references in this review. *Abbreviations*: CNS—central nervous system, Fe—ferrum, FPN1—ferroportin-1, DMT1—divalent metal transporter-1, HFE H63D—hemochromatosis H63D, TfC2—transferrin C2.

Increasing experimental and clinical evidence concerning iron metabolism support the idea that healthy aging processes, as well as neurological disorders, differ between women and men, suggesting the existence of different underlying mechanisms involved in the iron homeostasis and the pathogenesis of diseases (Figure 3) [16,17,18,19,20,21,22,161]. Age and sex are important co-factors to consider when establishing the differences between the pathological neurodegeneration from healthy aging. 

As for healthy aging, a sex-specific negative association was found between dietary iron intake and cellular aging markers. Iron intake showed deleterious effects on the peripheral blood leukocyte telomere length in women and on the number of mitochondrial DNA copies in men [162].

These effects of iron imbalance on genomic stability and cellular aging markers must be considered during dietary iron intake and iron supplementation [162]. In addition, brain iron concentration differs between older men and women, showing that women have lower total subcortical brain iron levels after expected menopause onset [17]. These findings indicate that age-related changes in estrogen levels may be a mediating factor of such associations.

However, the overall data that involve sex-related differences in iron dyshomeostasis and concomitant brain disorders are still limited and mainly concentrated on estrogen functions. Thus, we are far away from the actual understanding of what underlies these differences in iron metabolism during aging processes and therefore need further assessment.

### 5.1. Iron and Multiple Sclerosis

Investigations in animal model of MS, experimental autoimmune encephalomyelitis (EAE), showed worsening of clinical course in iron overloaded animals, which had an iron accumulation in CNS (brain and spinal cord) [16]. Although female IO rats developed symptoms earlier, male IO rats showed more severe clinical course and higher mortality rate, indicating the existence of sex-dependent mechanisms [163]. During the acute phase of EAE, female IO rats sequestered more iron in the liver and produced more ferritin than male EAE rats. Male rats, however, reacted on IO by higher production of oxidative stress markers, malondialdehyde and 4-hydroxynonenal, in the neural tissues and showed greater signs of plaque formation and gliosis in the spinal cord [16]. The data point to sexual dimorphism in mechanisms that regulate peripheral and brain iron homeostasis and imply that men and women during MS might be differentially vulnerable to exogenous IO. 

In patients with MS, iron content is elevated in deep grey matter structures and in the vicinity of lesions and reduced in the white matter [164]. Iron content is low in remyelinated plaques [164], suggesting that dynamic shuttling of iron continues through the MS disease process. This reveals that the iron dysregulation associated with MS is, in fact, a redistribution of iron between different areas of the brain [165]. Furthermore, quantitative MRI technique, i.e., quantitative susceptibility mapping suggests that altered deep grey matter iron is associated with the evolution of MS and on disability accrual, independent of tissue atrophy [166]. Excess iron enhances the generation of ROS, leading to myelin and neuron loss followed by demyelination and neurodegeneration [140]. 

Contrary to the belief that iron is harmful and invariably causes oxidative damage, it may paradoxically represent the key component of the entire antioxidant protection system of the oligodendrocyte. Namely, oligodendrocytes need iron for the extremely high energy requirements of producing and maintaining the complex myelin sheath [167], indicating that ID could seriously compromise the viability of these cells. Iron is also an important element for the maturation of oligodendrocyte progenitor cells (OPCs) into oligodendrocytes [168,169]. During remyelination, the OPCs are recruited to the MS lesions and differentiated into mature oligodendrocytes, which can further remyelinate the damaged axons. Considering the requirement for iron-containing enzymes in all these processes, iron levels in oligodendrocytes have an important influence on remyelination and neuronal repair. In the situation of reduced iron availability and iron-deficient oligodendrocytes, whether through global ID [170], impaired iron trafficking [171] or its export from astrocytes [11,172], it leads to reduced OPCs proliferation, disturbances in oligodendrocyte differentiation and following remyelination. Furthermore, OPCs are very sensitive to oxidation and the depletion of antioxidants such as glutathione, even more than mature oligodendrocytes [140]. This implies that these cells need antioxidant protection during patient relapses when there is an increased concentration of inflammatory mediators and ROS. Namely, iron is required for the production of ATP, which is essential for the synthesis of NADPH. NADPH is the reducing power of the cell [173] needed for the synthesis of lipids such as cholesterol, which are produced by oligodendrocytes for their membranes [173,174]. Heme cofactors in cytochrome P450 enzymes catalyze the essential hydroxylation reactions in the synthesis of cholesterol.

Furthermore, the hydroxylations that produce active vitamin D (1,25(OH)_2_ D_3)_ from cholesterol are carried out by a cytochrome P450 enzyme called CYP27B1 [175]. Oligodendrocytes express vitamin D_3_ receptors and respond to 1,25(OH)_2_ D_3_ [176]. Thus, the relevant cytochrome P450 enzyme with a heme group is synthesized only in the presence of sufficient iron. It is already well known that sufficient vitamin D is protective against MS [177] and is associated with improved clinical and MRI outcomes [178]. In addition, Vitamin D has been shown to decrease hepcidin, which inversely regulates serum iron level, and the optimal function of hepcidin may be predicated upon the adequate presence of vitamin D in the blood [179]. 

Recent data showed that elderly males suffer from a serious vitamin D deficiency compared to elderly females. Nevertheless, old age is an independent risk factor for vitamin D deficiency, so together with ID could worsen the MS progression [180]. A possible reason could be the connection of ID with the age-related changes associated with chronic inflammatory states [11].

The link between ID and obesity may also be of relevance in MS, since obesity may be a risk factor for MS [181]. Obesity is often present in elderly people, and ID in obese has been ascribed to chronic, low-grade inflammation [182]. There is sufficient evidence linking ID, even moderate one, with adverse health effects to justify the use of iron therapy. Therapy should be performed with caution to prevent the risk of high body iron stores and its detrimental effects on the brain [23]. 

Females exhibit lower levels of serum iron [183] and lower levels of brain iron compared to men from midlife to old age [184]. Pre-menstrual blood loss reduces serum iron levels in women and may contribute to sex differences in brain iron accumulation [185]. Namely, histological data suggest that lower serum iron levels may influence brain iron levels since anemia was found to reduce brain iron in people postmortem [186]. Men have shown higher iron concentrations in the cortical white matter and subcortical nuclei according to MRI images [183,185,187]. Changing of the sex steroids levels in post-menopause [188] may influence sex-related variations of brain iron levels as well [17]. However, a lack of sex-related differences in brain iron levels have also been reported [153]. 

On the other hand, although elderly patients with late onset of MS (LOMS) represent a growing minority of all patients with the diagnosis of MS, the LOMS (aged >65 years) has a worse prognosis, which is still subject to debate [189]. However, it could be associated with the facts that brain iron levels increase with age in healthy individuals [190] and that serum iron level is lowering at the same time [23]. Namely, it can be hypothesized that a shift of iron from the blood compartment to the brain compartment occurred due to iron dysregulation because of aging and leading to iron deposition due to excess iron in the brain. If the iron does play a role in the etiology of MS, it could be possible that some patients may need supplementation, and others’ attenuation of iron intake depending on their genetic background, making the individualized treatment approach for subgroups of MS. 

Another reason for taking into consideration the iron supplementation is the fact that MS patients eat a more limited diet, with a lower average of 31 nutrients, including zinc, thiamin, and iron, when compared with healthy controls. In a study by Armon-Omer et al., 2019 blood tests showed that MS patients had significantly lower iron levels, with the lowest measures in the severe MS group [191]. In conclusion, it is possible that inadequate iron levels (both low and high) may be harmful in MS. Iron excess might increase free radicals, which may elevate oxidative stress, while iron reduction could decrease immune system function and cause an energy deficit due to loss of mitochondrial membrane potential [11]. In addition, Armon-Omer and coworkers found lower dietary copper intake in the MS group, which is an essential cofactor for many oxidative enzymes and is necessary for iron absorption and transfer [191].

Some studies suggest that ID may play a role in MS disease progression as MS patients display clinical improvement upon iron supplementation. However, other studies indicate improved disease outcome in iron-limited MS patients [11]. These contradictory results may be due to differences in nutritional, biochemical and sex-related factors between subjects, requiring further investigation.

### 5.2. Iron and Stroke

Both iron deficiency and excess have been associated with stroke risk. Previous studies have found that both increase the risk of venous thromboembolism and carotid atherosclerosis [192]. Higher iron status is protective against some forms of the atherosclerotic disease but increases the risk of thrombosis related to stasis and is associated with increased stroke risk, in particular, cardioembolic stroke [15]. Further investigation is required to determine the precise mechanism of these effects. As previously reported, iron is a prooxidant cofactor associated with increased production of ROS. In the animal model, a moderate IO markedly accelerated thrombus formation, impaired vasoreactivity, and enhanced the production of ROS and systemic markers of oxidative stress [193]. Furthermore, the administration of ROS scavenger completely abrogates the iron load-induced thrombus formation, thus confirming that the iron accelerates thrombosis through a prooxidant mechanism [194]. 

Over the last few years, the association of ID and thrombophilia has also been increasingly studied. Various kinds of thrombotic diseases including central retinal vein occlusion, cerebral venous sinus thrombosis and carotid artery thrombus were observed to be associated with an ID. In addition, numbers of cases of embolic and ischemic stroke have been reported to be associated with the ID [195].

An increased plasma Tf level is often seen in patients suffering from ID anemia [196]. Beside the role of binding and transporting the plasma iron, Tf is also an important clotting regulator and an adjuster in the maintenance of coagulation balance, which modifies the coagulation cascade. In atherosclerosis, abnormally upregulated Tf interact with and potentiate thrombin/FXIIa and blocks antithrombin’s inactivation effect on coagulation proteases by binding to antithrombin, thus inducing hypercoagulability [197]. Furthermore, elevated Tf found in plasma or CSF of patients with ischemic stroke, ID anemia and venous thromboembolism interacts with clotting factors, suggesting that elevated Tf causes thromboembolic diseases [195]. Another consequence of ID that would be highly relevant to stroke pathogenesis is enhanced platelet aggregation. The platelet aggregation is enhanced as a response to serotonin (5 hydroxytryptophan, 5HT) in ID patients [198,199] because ID impairs the activity of the iron-containing platelet monoamine oxidase that metabolizes 5HT [200].

Gender plays an important role in the incidence of stroke. The overall incidence of stroke in men is estimated to be 33% higher than in women throughout most of the adulthood [201]. Findings from the prospective study in men from Kaluza et al., 2013 indicate that a high heme iron intake, particularly in normal-weight individuals, may increase the risk of stroke [202].

The epidemiology of stroke changes as women age and coincide with the loss of estrogen after menopause [18]. Furthermore, elderly women have more severe strokes, poorer recovery, and greater long-term disability [203], compared with men of the same age.

Estrogen is an immunomodulatory and neuroprotective agent with a suppressive action on inflammation [204]. It is known that postmenopausal women have higher levels of circulating TNF-α [205], which is involved in many neurodegenerative diseases [206]. 

Research evidence showed that TNF is beneficial in injury repair, but high levels of TNF can be neurotoxic [207]. Physiological levels of estrogen appear to attenuate TNF expression [208] while estrogen deficiency (as in postmenopausal period) represents a loss of this attenuation with a subsequent increase in TNF expression [18]. An increase of inflammatory agents in postmenopausal period could be associated with the higher hepcidin production and subsequent decrease in iron absorption, which could be an additional potential risk factor for ischemic stroke in women [205,209]. 

On the other hand, in the study from Miller [210], hormone replacement therapy was associated with lower iron stores in post-reproductive women in the absence of uterine blood loss, indicating potential homeostatic hormonal control of the iron status. Namely, higher serum iron in post-menopause has traditionally been attributed to reduced menstrual bleeding and lack of iron loss that women experience with menopause, in addition to estrogen deficiency [210]. With findings from studies on the levels of ferritin and sex hormones, it can be concluded that as women age, their serum levels of estrogen decrease, while serum ferritin levels increase [211]. This is probably due to an increase in the iron regulatory hormone hepcidin since elevated levels of estrogen usually reduce hepcidin synthesis [209], which regulates ferritin. Before the onset of menopause hepcidin levels in women are nearly 50% lower than in males of corresponding ages. After the menopause, hepcidin levels tend to be similar in both sexes [212,213] or slightly increased in men [214]. These results demonstrate a negative correlation between ferritin and estrogen levels during the menopausal transition period [215]. On the other hand, a synchronized pattern of changes in ferritin and testosterone levels was observed in men. Namely, as the men age, ferritin levels decrease gradually following »andropause« [216]. These results indicate that iron accumulation was a common process in aging women (but without the IO), which may account for the observed differences between genders in the incidence of the aging disorder, including the stroke [217].

Among older men with low testosterone levels, testosterone treatment can increase the serum iron levels and correct ID anemia [218]. Also, data are showing significant overlap between the testosterone administration and IO [219].

Furthermore, as the body is unable to eliminate excess iron, a negative feedback mechanism that allows iron to inhibit testosterone production to maintain body iron homeostasis is proposed [219]. The body iron stores can be regulated by testosterone, and vice versa, the testosterone may be reciprocally regulated by iron. Crosstalk between testosterone and iron has significant implications in testosterone deficiency and therapy. Additionally, the regulation of testosterone by iron may indicate a significant role of iron in the development of the hypogonadotropic hypogonadism in aging and chronic disease in men [219,220,221]. In a study from Jeppesen et al., 1996 both total and free testosterone were significantly inversely associated with stroke severity, and total testosterone was significantly inversely associated with infarct size [222]. Furthermore, a study from Zeller et al., 2018 suggested that low testosterone levels are associated with increased risk of future ischemic stroke in men [19]. The possible explanation lies in an increased level of hepcidin, which is inversely regulated by testosterone [223] and leads to ID anemia with greater susceptibility to stroke. 

These data also suggest another mechanism regarding stroke pathogenesis, which includes initially increased serum iron level that negatively regulates testosterone level in men and promotes susceptibility to stroke. However, low testosterone level is probably not an independent risk factor for stroke, especially in older men. Low testosterone level is more likely to be found in overweight or obese, which is significantly associated with cardiovascular risk factors, such as diabetes, high blood pressure and high cholesterol. Namely, adipose tissue is able to control several functions of the testis through its products secreted in the bloodstream (e.g., leptin, adipocytokines), which have a negative impact on Leydig cell’s function and testosterone secretion [224].

On the other hand, testosterone exerts a significant inhibitory effect on adipose tissue formation and the expression of various adipocytokines, such as leptin, TNF-*α*, IL-6, and IL-1, whereas a low testosterone level correlates with increased expression of markers of inflammation [225]. Furthermore, low chronic inflammation due to the excess adipose tissue upregulates hepcidin, which lowers the iron serum levels and its absorption [163,226], so eventually serum iron levels will decrease and subsequently lead to ID, present at the same time with the low testosterone level.

### 5.3. Iron and Parkinson’s Disease

Dopaminergic neurons in the *substantia nigra* are highly vulnerable to stress conditions, compared to other neuronal types. Different factors seem to contribute to oxidative stress in PD, including IO, neuroinflammation and aging [14,206,227]. 

Several researches have documented an increase in total iron concentration in the *substantia nigra* in the most severe cases of PD, but no changes were found in milder cases [228,229]. Increased iron concentrations in the *substantia nigra* might result from mutations in genes important for iron transport and binding [230] or from peripheral iron influx through a damaged and discontinued BBB via the Permeability-glycoprotein [165]. Furthermore, the ability of the lysosome to participate in autophagy becomes slower with age, resulting in an increase of non-protein »garbage« within the cells, especially in age-related diseases like PD [227].

Accumulated iron increases protein aggregation via enhanced generation of ROS and oxidative stress [231]. As already mentioned, iron accumulation in the *substantia nigra* in PD patients was confirmed, but the studies on alteration of iron levels in blood and CFS reported inconsistent results. Blood levels of iron did not differ significantly between PD patients and the controls, but CSF iron levels tended to be lower in PD patients compared to the controls [232]. 

Increasing experimental and clinical evidence supports the idea that PD differs between women and men [21]. Although males exhibit greater susceptibility, most studies concentrate on the neuroprotective effects of estrogens in females. It was shown that men and women experience the disease differently, suggesting different mechanisms involved in the pathogenesis of the disease [21].

Although the sexual dimorphism in brain mitochondria has been proven [20], there are few recent studies that have dealt with other possible causes of sex-related differences. A recent neuromelanin imaging study found a larger neuromelanin-rich volume in the women *substantia nigra* compared with men older than 47 years, suggesting that this difference may be the underlying cause of the high male-to-female ratio of the PD prevalence [233]. Furthermore, experimental and epidemiological evidence suggest that estrogens play a regulatory role in brain iron metabolism [223,234]. The striatum of male mice showed greater susceptibility to iron accumulation than female [235]. A study conducted on humans showed that at the same serum iron concentrations, women had a lower probability of having PD [236]. Recently, several different pathways related to estrogen effects on iron metabolism in both sexes were found. GPER1 (G Protein-Coupled Estrogen Receptor 1) mediates the suppressive effects of estrogen on IO-induced autophagy in males, while estrogen receptor suppresses induced lipid peroxidation in females [237]. Furthermore, the results from the study of Xu and coworkers, showed that estrogen regulates differently the iron metabolism in astrocytes and neurons, i.e., increases the expression of iron exporter FPN1 and iron importer DMT1 by inducing HIF-1α in astrocytes, whereas decreased expression of IRP-1 may account for the decreased DMT1 and increased FPN1 expression in neurons [238].

Nutrition plays an important role in both neuroprotection and neurodegeneration, although there are many conflicting results. A recent epidemiological study found that intake of meat was inversely associated with PD risk in women [239]. Usually, the higher incidence of mortality, cardiovascular diseases, and diabetes is associated with higher meat consumption [240,241]. Furthermore, a positive correlation between red meat consumption and PD may be explained by the heme content that increases intracellular iron concentrations and subsequent ROS production, contributing to iron deposits and cell damage. In this context, iron intake from dietary nutrients may be related to a higher risk for PD [242]. However, blood donations, which can decrease systemic iron stores, do not lower the risk of PD [243]. On the other hand, conflicting results from Miyake et al., 2011 study showed that higher intake of iron could be associated with neuroprotection in PD [244].

In contrast to higher iron level content in the body, certain authors disagree about the implication of ID on the pathogenesis of PD [165]. ID anemia and low Hb have also been associated with PD i.e., with increased risk and disease severity [245]. Furthermore, it has been documented that PD patients exhibit lower ferritin, TIBC, and serum iron levels [245]. A positive correlation between anemia and PD was found in a recent large study of 86,334 newly diagnosed anemic patients. The study suggested that de novo anemic patients may develop PD 4 or more years after the initial diagnosis of anemia [246] and a higher risk for PD was independent of iron supplementation [247].

On the other hand, the meta-analysis from Mariani et al., 2013 showed that there is no difference in iron between PD patients and healthy controls [248].

Possible reasons for these discrepancies may be that the total amount and the serum iron in patients with PD did not change, but the distribution of iron changed [249], i.e., iron aggregated in the *substantia nigra*. Besides, this heterogeneity can manifest as highly variable iron metabolism due to sex-specific differences [250], which could lead to inconsistent results. However, epidemiological studies have not identified any sex-specific factors in the risk of developing PD among anemic patients. 

We must take into account that besides the dysregulated iron metabolism, the presence of anemia in PD could also be an indicator of poor absorption of other nutrients [251].

### 5.4. Iron and Alzheimer’s Disease

Alzheimer’s disease is the most common cause of dementia characterized by short term memory loss and a progressive decline in cognitive and motor functions [165]. In neurodegenerative diseases including AD, where age is the major risk factor, iron dyshomeostasis coincides with neuroinflammation, abnormal protein aggregation, neurodegeneration, and neurobehavioral deficits. Disruption of iron homeostasis in the brain, both deficiency and overload, can affect neurophysiological mechanisms, cognition, and social behavior, which eventually contributes to the development of a diverse set of neuro-pathologies. Using MRI, it was found that the iron content in the brains of AD patients was significantly increased [252].

Whether the iron accumulation present in neurodegenerative diseases is a primary event or a secondary effect of the disease is unclear. However, it is for sure that aging is the major risk factor for age-related iron accumulation and neurodegeneration [13]. Iron seems to promote both deposition of amyloid-β protein and oxidative stress, which is associated with the plaques [7,253]. In contrast, some argued that, by binding iron, Aβ-protein might protect the surrounding neurons from oxidative stress [254].

Decreased antioxidant defenses and mitochondrial dysfunction present in elderly can allow the release of excessive iron [255]. This can cause pathological IO, resulting in cellular damage that is considered to be a contributing factor in neurodegenerative diseases more prevalent with aging, such as AD [165]. The results from recent studies showed that elevated ferritin in CSF was associated with poor cognitive function and probably can be used as a biomarker to measure the progression of mild cognitive impairment and early AD [256,257].

Brain iron increases with age and is abnormally elevated early in the disease [258]. Higher brain iron levels were associated with male gender and the presence of allelic variants in genes encoding for iron metabolism proteins (hemochromatosis H63D (HFE H63D) and transferrin C2 (TfC2)). This genotype effect was not observed in women, who had lower iron content in the brain than men [22,258]. The results showed worse verbal-memory performance associated with higher hippocampal iron deposition in men but not in women, independent of gene status. Furthermore, independent of gender, worse verbal working memory performance was associated with higher basal ganglia iron in the non-carrier for HFE H63D/TfC2 gene variant but not in the carrier. These results suggest that in healthy older individuals, increased deposits of iron in vulnerable gray matter regions may negatively impact memory functions and could represent a risk factor for faster cognitive decline [22].

Furthermore, it was shown in many in vivo and in vitro studies that iron metabolism is integrally involved in the regulation of glutamate metabolism and vice versa. The results from Burger et al., 2020, showed association between iron metabolism and glutamate concentration in female, suggesting stronger regulatory control between iron and glutamate metabolism than in men [259]. 

Aging is associated with a gradual decline in sex hormone levels in men and women, together with a deterioration in general health, mood, and cognitive abilities [260,261]. Sex hormones are also protective in keeping amyloid down, while depleted estrogen and testosterone levels result in a massive rise in this toxic protein in the brain [260,262]. 

Estrogens are considered as potent neuroprotectants and the best-studied in vitro and in vivo class of drugs for potential use in the prevention of AD [260]. In cell-free systems, estrogens inhibit iron-induced lipid peroxidation [263,264]. However, this effect was not found with testosterone [264]. 

In elderly, anemia or abnormal Hb concentrations are associated with higher morbidity and mortality, and with an increased risk for dementia and rapid cognitive decline [265].

The studies in elderly populations have confirmed that anemia and lower serum Hb were associated with a twofold increased risk for developing AD over approximately 3 years [266,267]. The findings from Carlson, et al. 2008 suggest a role of neonatal ID in dysregulation of genes that may set the stage for long-term AD and that this may occur through a histone modification mechanism [268]. 

It is important to have in mind that iron supplementation improves attention and concentration irrespective of baseline iron status [269]. However, as said before, excess of iron mediates the oxidative stress and causes neuronal disorders and neurodegeneration. In addition, it is thought that imbalance in iron homeostasis is a precursor to AD as well, thus it is strongly suggested that older people should be careful with diets excessive in iron.

## 6. Conclusions

Older age is associated with increased risk of ID, elevated body iron stores and increased brain iron levels [23]. Inadequate iron supply, which often accompanies aging, leads to cerebral hypoxia [138], insufficient neurotransmitter synthesis [13], impaired myelination [139], and consequently to poorer cognition, cognitive decline, and dementia [141]. On the other hand, brain iron accumulation is considered as a hallmark of aging [146] and it is associated with the progressive imbalance between antioxidant defenses and intracellular generation of ROS [147]. This increases susceptibility of aged brain to diseases and thus makes aging a major risk factor for neurodegenerative diseases development [14]. Therefore, it would be very important for the future research to determine the exact cellular and molecular mechanisms related to perturbations in iron metabolism in the aging brain to distinguish between physiological and pathological aging and find possible therapeutic targets for neurodegenerative diseases.

To counteract the ID during aging, one should certainly consider iron supplements recommended by a physician to correct the anemic state.

However, it should be noted how this supplementation may not be warranted for healthy elderly people consuming a balanced diet. In contrary, it could be detrimental for those who are homozygous or heterozygous for the HFE mutations, since recent studies showed that even moderate increases in body iron may increase the risk for body disorders including neurological ones [270], or cause irreparable damage to the brain neurons [137,271]. Because of that, older people should be careful consuming a high-iron content diet as well. 

The major unknown is still the sex-related differences in iron metabolism that come with aging. Increasing experimental and clinical evidence support the idea that neurological disorders differ between women and men, suggesting the existence of different underlying mechanisms involved in their pathogenesis [16,17,18,19,20,21,22]. However, we are still far away from the actual understanding of what underlies these differences. We need a better understanding of the underlying mechanisms of how sex hormones can influence the iron metabolism and further, the development of neurological disorders. New insights into aging processes, which include the impact of sex hormones on iron metabolism as well, could enlighten the understanding of these differences during aging.
